# High Viral Loads of Epstein-Barr Virus DNA in Peripheral Blood of Patients with Chronic Lymphocytic Leukemia Associated with Unfavorable Prognosis

**DOI:** 10.1371/journal.pone.0140178

**Published:** 2015-10-13

**Authors:** Ewelina Grywalska, Jacek Roliński, Marcin Pasiarski, Izabela Korona-Glowniak, Maciej Maj, Agata Surdacka, Agnieszka Grafka, Agnieszka Stelmach-Gołdyś, Michał Zgurski, Stanisław Góźdź, Anna Malm, Piotr Grabarczyk, Elżbieta Starosławska

**Affiliations:** 1 Department of Clinical Immunology and Immunotherapy, Medical University of Lublin, Lublin, Poland; 2 St. John’s Cancer Center, Lublin, Poland; 3 Department of Hematology, Holycross Cancer Center, Kielce, Poland; 4 Department of Pharmaceutical Microbiology, Medical University of Lublin, Lublin, Poland; 5 Department of Oncology, Holycross Cancer Center, Kielce, Poland; 6 Faculty of Health Sciences, Jan Kochanowski University, Kielce, Poland; 7 Department of Immunohematology, Institute of Hematology and Transfusion Medicine, Warsaw, Poland; The University of North Carolina at Chapel Hill, UNITED STATES

## Abstract

Epstein-Barr virus (EBV) is a ubiquitous *γ*-herpesvirus that infects more than 90% of the world population. The potential involvement of EBV in the clinical course of chronic lymphocytic leukemia (CLL) remains unexplained. The aim of this study was to determine whether EBV-DNA load in the peripheral blood mononuclear cells (PBMCs) of CLL patients may influence heterogeneity in the course of the disease. The study included peripheral blood samples from 115 previously untreated patients with CLL (54 women and 61 men) and 40 healthy controls (16 women and 24 men). We analyzed the association between the EBV-DNA load in PBMCs and the stage of the disease, adverse prognostic factors, and clinical outcome. Detectable numbers of EBV-DNA copies in PBMCs were found in 62 out of 115 CLL patients (53.91%). The EBV-DNA copy number/*μ*g DNA was significantly higher in patients who required early implementation of treatment, presented with lymphocyte count doubling time <12 months, displayed CD38-positive or ZAP-70-positive phenotype, and with the del(11q22.3) cytogenetic abnormality. Furthermore, the EBV-DNA copy number/*μ*g DNA showed significant positive correlation with the concentrations of lactate dehydrogenase (LDH) and beta-2-microglobulin. We have shown that in CLL patients, higher EBV-DNA copy number predicted shorter survival and shorter time to disease progression, and it was associated with other established unfavorable prognostic factors. This suggests that EBV may negatively affect the outcome of CLL.

## Introduction

Epstein-Barr virus (EBV), also referred to as human herpesvirus 4 (HHV-4), is the first identified human virus with documented involvement in carcinogenesis [1]. Many cells of the non-Hodgkin lymphomas (NHL) show the presence of the monoclonal form of EBV genome (i.e. the EBV-positive phenotype). This finding points to the origin of the malignancy from a single infected cell and involvement of EBV in its pathogenesis [2, 3]. A number of hypotheses have been proposed to explain the etiology of NHL in individuals from the general population with inadequately controlled EBV infection. One postulated mechanism is the EBV-induced proliferation of B lymphocytes and a resultant increase in the number of B-cells at risk of oncogenic mutations and clonal expansion [4].

Despite its chronic character by definition, chronic lymphocytic leukemia (CLL) is characterized by marked heterogeneity [5, 6]. Only 30% of patients survive up to 10–20 years after diagnosis [7]. The remaining CLL patients develop terminal phase within 5–10 years, despite mild onset of the disease. The individuals with the aggressive form of CLL survive no more than 2–3 years after diagnosis [8]. The reasons for such heterogeneous natural history of the condition remain unclear.

Potential involvement of EBV in the clinical course of CLL is still unexplained. Latent EBV infection is controlled by a cell-mediated immune response in healthy carriers. This immune response is impaired in CLL patients and might result in poor control of reactivation and replication of the virus. Since EBV may activate B cells, stimulate their proliferation, and inhibit their apoptosis, we hypothesized that it could contribute to unfavorable clinical course of CLL and may be one of the reasons for the observed disease heterogeneity.

The aim of this study was to define a role of EBV in the etiopathogenesis of CLL. The detailed objectives included the determination of the EBV-DNA copy number in mononuclear cells and isolated B lymphocytes from peripheral blood of CLL patients and healthy individuals and the analysis of association between this parameter and the established prognostic factors, stage of the disease, and its clinical manifestation.

## Methods

### Characteristics of CLL patients and healthy volunteers

The study included peripheral blood samples from 115 previously untreated patients with CLL (54 women and 61 men). The control group comprised 40 healthy subjects (16 women and 24 men). Neither the CLL patients nor the controls used immunomodulating agents or hormonal preparations, showed signs of infection within at least 3 months prior to the study, underwent blood transfusion, or presented with autoimmune condition or allergy. Moreover, none of the controls had a history of oncological therapy or prior treatment for tuberculosis or other chronic conditions that could be associated with impaired cellular or humoral immunity.

The diagnosis of CLL was established on the basis of diagnostic criteria included in the IWCLL guidelines of the American National Cancer Institute (NCI) [9, 10]. Detailed characteristics of patients and controls are presented in Table 1 and Table 2.

**Table 1:** Characteristics of chronic lymphocytic leukemia (CLL) patients and univariable analysis for time to first treatment.

**Parameter**	**Median (range)**	**Mean±SD**	**Hazard ratio (95%CI)**	**P value**
Age (years)	64 (38–89)	63.27±9.73	0.99 (0.97–1.03)	0.92
Observation period (months)	33 (10.50–80)	34.05±13.39	-	-
Lymphocyte doubling time (months)	8.75 (0.25–94)	14.70±17.02	0.97 (0.95–0.99)	0.012
Time from diagnosis to initiation of treatment (months)	13 (0.25–72)	17.88±15.86	-	-
White blood cell count, WBC (G/L)	30.19 (9.70–128)	37.09±22.94	0.99 (0.98–1.00)	0.29
Lymphocytosis (G/L)	24.55 (6.16–124)	31.09±22.16	0.99 (0.98–1.00)	0.29
Hemoglobin (g/dL)	13 (9–16.8)	13.05±1.69	0.78 (0.65–0.94)	0.011
Platelets (G/L)	172 (57–309)	174.77±58.36	1.00 (0.99–1.00)	0.28
Lactate dehydrogenase, LDH (U/L)	298 (96–955)	306.63±133.02	1.00 (1.00–1.004)	0.018
Beta-2 microglobulin, B2M (mg/L)	2.53 (1.10–8.14)	2.83±1.24	1.40 (1.12–1.74)	0.0028
IgA (g/L)	1.21 (0.13–5.40)	1.44±1.06	0.91 (0.69–1.22)	0.54
IgG (g/L)	8.85 (3.18–19.41)	9.15±3.64	1.05 (0.96–1.15)	0.28
IgM (g/L)	0.5 (0.04–3.13)	0.66±0.52	1.37 (0.75–2.47)	0.30
EBV-DNA copies/μg DNA	18.0 (0.0–8957.7)	428.47±1180.4	1.0002 (1.00004–1.0003)	0.013
CD19+CD25+ cells [%] *	54.75 (6.72–93.87)	54.96±20.50	1.03 (1.01–1.05)	0.0001
CD19+CD69+ cells [%] *	28.73 (0.55–78.82)	31.06±19.53	1.02 (1.00–1.04)	0.0083
CD3+CD25+ cells [%] *	18.37 (0.63–59.38)	19.89±14.39	1.019 (0.998–1.041)	0.080
CD3+CD69+ cells [%] *	3.07 (0.06–29.32)	4.64±4.70	1.07 (1.02–1.13)	0.0090
CD19+CD5+ZAP-70+ cells [%]*	14.19 (0.18–91.4)	16.30±12.80	1.012 (0.99–1.032)	0.22
CD19+CD5+CD38+ cells [%]*	9.01 (0.18–91.4)	22.95±25.85	1.02 (1.01–1.03)	0.00031
CD19+CD5+CD23+ cells [%] *	80.38 (46.05–95.88)	78.69±12.55	1.02 (1.00–1.05)	0.034
Anti-EBV EA IgA	5.20 (1.0–580.38)	30.50±86.20	1.004 (0.999–1.009)	0.15
Anti-EBV EA IgG	13.58 (1.92–381.1)	54.38±86.17	1.004 (1.00–1.007)	0.014
Anti-EBV EA IgM	3.81 (0.79–62.70)	5.96±7.9	1.001 (0.98–1.027)	0.89
Anti-EBV EBNA-1 IgA	5.43 (1.24–78.51)	8.86±10.98	1.011 (0.99–1.035)	0.33
Anti-EBV EBNA-1 IgG	65.76 (22.41–1701.82)	146.53±253.94	1.001 (1.0003–1.002)	0.008
Anti-EBV EBNA-1 IgM	5.52 (0.89–20.72)	6.34±4.22	1.017 (0.96–1.082)	0.60
Anti-EBV VCA IgA	6.95 (1.87–50.90)	10.55±8.75	1.027 (0.99–1.061)	0.099
Anti-EBV VCA IgG	183.74 (23.80–358.05)	180.89±91.26	1.006 (1.002–1.010)	0.0062
**Parameter**	**Number of patients**	**Percentages (%)**	**Hazard ratio (95%CI)**	**P value**
Rai Stage				
0	48	41.74%	II-IV v 0–1:0.84 (0.46–1.53)	0.57
I	27	23.48%		
II	30	26.09%		
III	2	1.74%		
IV	8	6.96%		
Binet classification				
A	48	41.74%	baseline	
B	57	49.57%	1.02 (0.48–2.16)	0.96
C	10	8.69%	1.24 (0.47–3.27)	0.68
Splenomegaly				
Yes	35	30.43%	1.87 (1.02–3.44)	0.043
No	80	69.57%	baseline	
Hepatomegaly				
Yes	19	16.52%	1.33 (0.68–2.60)	0.41
No	96	83.48%	baseline	
Doubling lymphocytosis in observation period				
Yes	68	59.13%	2.28 (0.88–5.95)	0.09
No	47	40.87%	baseline	
Length of lymphocyte doubling time				
shorter than 6 months	22	32.35 (19.13% among all patients)		
6–12 months	24	35.30 (20.87% among all patients)		
longer than 12 months	22	32.35 (19.13% among all patients)		
Treatment onset in observation period				
Yes	45	39.13%	5.66 (0.76–42.08)	0.09
No	70	60.87%	baseline	
Outcome				
Complete remission after treatment	10	21.28% (8.69% among all patients)		
Partial remission after treatment	5	10.64% (4.35% among all patients)		
Progressive disease	24	51.06% (20.87% among all patients)		
CLL-associated deaths	8	17.02% (6.96% among all patients)		
ZAP-70 (cut-off 20%)				
Positive	41	35.65%	1.35 (0.74–2.46)	0.33
Negative	74	64.35%	baseline	
CD38 (cut-off 30%)				
Positive	43	37.39%	2.82 (1.42–5.62)	0.0032
Negative	72	62.61%	baseline	
Cytogenetic abnormalities				
del(13q14.3) only	13	11.30%	baseline	
del(17p13.1) only	6	5.22%	1.65 (0.39–6.97)	0.50
trisomy 12 only	4	3.48%	10.09 (1.60–63.79)	0.014
del(11q22.3) only	20	17.39%	1.60 (0.48–5.38)	0.44
double del:	16	13.91%	1.69 (0.47–6.02)	0.42
del(13q14.3) and trisomy 12	6	5.22%		
del(13q14.3) and del(11q22.3)	5	4.35%		
del(17p13.1) and del(11q22.3)	2	1.74%		
del(17p13.1) and del(13q14.3)	2	1.74%		
del(17p13.1), del(11q22.3) and del(13q14.3)	1	0.87%		
Negative	56	48.69%	0.66 (0.22–1.98)	0.46
Thrombocytopenia (platelets < 100 G/L)				
Yes	9	7.83%	1.05 (0.47–2.39)	0.90
No	106	92.17%	baseline	
LDH elevated above the reference ranges (81–234 U/L)				
Yes	79	68.7%	1.45 (0.64–3.26)	0.37
No	36	31.3%	baseline	
B2M elevated above the reference ranges (1.09–2.53 mg/L)				
Yes	57	49.57	1.58 (0.81–3.09)	0.17
No	58	50.43	baseline	
EBV				
Positive	62	53.9%	7.26 (3.05–17.31)	< 0.0001
Negative	53	46.1%	baseline	
Presence of other cancer				
Yes	17	14.8%	0.84 (0.43–1.67)	0.62
No	98	85.2%	baseline	
Anti-EBV EA IgA				
Positive	34	29.6%	1.29 (1.04–3.62)	0.036
Negative	81	70.4%	baseline	
Anti-EBV EA IgG				
Positive	50	43.5%	1.68 (0.92–3.05)	0.091
Negative	63	56.5%	baseline	
Anti-EBV EA IgM				
Positive	18	15.7%	1.72 (0.84–3.49)	0.14
Negative	97	84.3%	baseline	
Anti-EBV EBNA-1 IgA				
Positive	35	30.4%	0.92 (0.52–1.65)	0.79
Negative	80	69.6%	baseline	
Anti-EBV EBNA-1 IgG				
Positive	115	100%	-	-
Negative	0	0%	-	-
Anti-EBV EBNA-1 IgM				
Positive	25	21.7%	1.70 (0.89–3.26)	0.11
Negative	90	78.3%	baseline	
Anti-EBV VCA IgA				
Positive	21	18.3%	1.45 (0.72–2.89)	0.29
Negative	94	81.7%	baseline	
Anti-EBV VCA IgG				
Positive	115	100%	-	-
Negative	0	0%	-	-
Hypogammaglobulinemia IgA				
Yes	32	27.8%	1.81 (0.91–3.62)	0.091
No	83	72.2%	baseline	
Hypogammaglobulinemia IgG				
Yes	41	35.7%	1.23 (0.65–2.33)	0.52
No	74	64.3%	baseline	
Hypogammaglobulinemia IgM				
Yes	40	34.8%	1.56 (0.79–3.08)	0.20
No	75	65.2%	baseline	

* in the peripheral blood

**Table 2:** Characteristic of control group.

Parameter	Number of controls	Percentages (%)
Age (years)		
Median 65.5 (range: 53–79), mean 64.50±7.15		
White blood cell count (G/L)		
Median 7.02 (range: 4.23–9.63), mean 7.01±1.44		
Lymphocytosis (G/L)		
Median 2.62 (range: 1.39–4.16), mean 2.65±0.82		
Hemoglobin (g/dL)		
Median 14.35 (range: 12.5–16.9), mean 14.3±1.19		
Platelets (G/L)		
Median 281.5 (range: 186–360), mean 274±49.49		
Lactate dehydrogenase LDH (U/L)		
Median 155 (range: 111–209), mean 157±27.96		
Beta-2 microglobulin, B2M (mg/L)		
Median 1.68 (range: 1.06–2.3), mean 1.63±0.39		
IgA (g/L)		
Median 2.56 (range: 0.92–3.92), mean 2.39±0.84		
IgG (g/L)		
Median 12.79 (range: 10.06–15.47), mean 12.71±1.4		
IgM (g/L)		
Median 1.61 (range: 1.17–2.19), mean 1.66±0.31		
Splenomegaly		
Yes	0	0%
No	40	100%
Hepatomegaly		
Yes	0	0%
No	40	100%
Thrombocytopenia (platelets<100 G/L)		
Yes	0	0%
No	20	100%
LDH elevated above the reference ranges (81–234 U/L)		
Yes	0	0%
No	20	100%
B2M elevated above the reference ranges (1.09–2.53 mg/L)		
Yes	0	0%
No	20	100%

This study was approved by the Ethics Committee of the Medical University of Lublin (decision no. KE-0254/227/2010). Written informed consent was obtained from all patients with respect to the use of their blood for scientific purposes.

### Examined material

Peripheral blood (20 mL) from the basilic vein of CLL patients and healthy controls was collected into EDTA-treated tubes (15 mL) and into tubes containing clot activator (5 mL) (aspiration and vacuum systems Sarstedt, Germany). Immediately after collection, the samples were used for immunophenotyping of lymphocytes, isolation of mononuclear cells for the EBV-DNA copy number determination, serum collection for the determination of specific anti-EBV antibodies concentration, and cytogenetic studies.

### Isolation of mononuclear cells and serum

Peripheral blood was diluted with 0.9% buffered saline (PBS) without calcium (Ca^2+^) and magnesium (Mg^2+^) (Biochrome AG, Germany) in 1: 1 ratio. The diluted material was built up with 3 mL of Gradisol L (specific gravity 1.077 g/ml; Aqua Medica, Poland), and centrifuged in a density gradient at 700 × g for 20 min. The obtained fraction of peripheral blood mononuclear cells (PBMCs) was collected with Pasteur pipettes and washed twice in PBS without Ca^2+^ and Mg^2+^ for 5 min. Subsequently, the cells were suspended in 1 mL of PBS without Ca^2+^ and Mg^2+^, and either counted in the Neubauer chamber or tested for viability with trypan blue solution (0.4% Trypan Blue Solution, Sigma Aldrich, Germany). Viability below 95% disqualified the cells from further analyses.

Serum was obtained from the samples collected into the tubes containing clot activator, aliquoted, and stored at –80°C for enzyme-linked immunosorbent assay (ELISA) test.

### Isolation of DNA and determination of the EBV copy number

DNA from 5 × 10^6^ PBMCs was isolated manually with the QIAamp DNA Blood Mini Kit (QIAGEN, Germany). The procedure for isolation followed the manufacturer’s protocol, with a modified volume of DNA elution. Concentration and purity of the isolated DNA were verified with the BioSpec-nano spectrophotometer (Shimadzu, Japan). The EBV-DNA copy number in PBMCs was determined with the ISEX variant of the EBV PCR kit (GeneProof, Czech Republic). Qualitative and quantitative diagnostics of EBV was performed using the Real Time Polymerase Chain Reaction (RT-PCR). Specific conservative DNA sequence of a single-copy gene for the EBV nuclear antigen 1 (EBNA-1) was amplified in the course of the PCR process. The number of viral DNA copies/*μ*L of the eluent was adjusted for the efficiency of the DNA isolation process, and then it was expressed as the viral DNA copy number/*μ*g DNA. All the samples were examined in duplicates. A negative control, i.e. the pure buffer used for DNA elution, was amplified in every case. As the sensitivity of the system amounts to 10 copies/μL, all the samples with the EBV-DNA copy number below this detection threshold were considered EBV-negative [EBV(–)]. The PCR was performed with the 7300 Real Time PCR System (Applied Biosystems). The reaction was conducted on MicroAmp® Optical 96-Well Reaction Plates (Life Technologies) with MicroAmp® Optical Adhesive Film (Life Technologies).

### Assessment of activated T and B cells

A standard, whole-blood assay with erythrocyte cell lysis was used for preparing the peripheral blood specimens. The cells were phenotypically characterized by incubation (20 min in the dark at room temperature) with a combination of relevant fluorescein isothiocyanate (FITC)-, phycoerythrin (PE)-, and CyChrome-labelled monoclonal antibodies (MoAbs). Immunofluorescence studies were performed using a combination of the following MoAbs: CD3 PE, CD19 PE, CD5 FITC/CD19 PE, CD4 PE, CD8 PE, CD8 FITC/CD4 PE, CD25 CyChrome, and CD69 CyChrome, purchased from BD Biosciences (USA). Finally, cells were washed and analyzed by flow cytometry, performed on a BD FACSCalibur System. Five data parameters were acquired and stored: linear forward and side scatter (FSC, SSC), log FL-1 (FITC), log FL-2 (PE), and log FL-3 (PE-Cy5). For each analysis, 20,000 events were acquired and analyzed using CellQuest Pro software. Isotype-matched antibodies were used to verify the staining specificity and as a guide for setting the markers to delineate positive and negative populations. Mean fluorescence intensity (MFI) and the percentage of cells expressing surface markers were analyzed.

### Analysis of CD38 and ZAP-70 expression in CLL cells

CLL cells were stained for CD38 antigen and ZAP-70 protein expression (as described previously by Hus et al. [11]) and analyzed using flow cytometry. The cut-off point for CD38 and ZAP-70 positivity in leukemic cells was ≥30% and ≥20%, respectively.

### I-FISH analysis

PBMCs were cultivated for 24 hours in RPMI 1640 medium without mitogen stimulation. After hypotonic treatment and methanol—acetic acid 3: 1 fixation, cell suspensions were dropped onto microscopic slides and used directly for I-FISH. The commercially available Vysis probes (Abbott Molecular Europe, Wiesbaden, Germany) LSI ATM SpectrumOrange/CEP 11 SpectrumGreen Probe, and LSI TP53 SpectrumOrange/CEP 17 SpectrumGreen Probe were used. At least 200 nuclei were analyzed for each probe. The cut-off levels for positive values for normal controls were 2.5% (mean ± SD).

### Anti-EBV immunoassay

Commercial enzyme-linked immunosorbent assay (ELISA) kits, purchased from IBL International (Germany) for a quantitative determination of specific anti-EBV antibodies in human serum were used. Protocols followed were in accordance with the manufacturer’s recommendations. The ELISA Reader Victor TM3 (PerkinElmer, USA) was used.

### Statistical analysis

Normal distribution of continuous variables was tested using the Shapiro-Wilk test. Statistical characteristics of the continuous variables were presented as medians, minimum and maximum values, as well as arithmetic means and their standard deviations (SD). The Student *t*-test was used for independent variables, and the Mann-Whitney *U*-test was used for intergroup comparisons. The power and direction of relationships between pairs of continuous variables were determined on the basis of the values of Spearman’s coefficient of rank correlation (*R*). The distributions of discrete variables in the studied groups were compared with the Pearson’s Chi-square test or the Fisher’s exact test. The survival curves were constructed with the Kaplan-Meier method, and the proportions of survivors within the studied groups were compared with the log-rank test. Univariable and multivariable Cox proportional hazard regression models were used to determined association between patients characteristics and time to first treatment. For the multivariable model, stepwise variable selection was used. Significant variables (p<0.1) were tested for inclusion in the regression models, and nonsignificant variables were removed sequentially until only those significant at p<0.05 remained.

Receiver operating characteristic (ROC) curves were generated for significant predictor variables of EBV(+) CLL patients. Areas under the ROC curves (AUCs) were calculated for each parameter and compared. All the calculations were carried out with Statistica 10 (StatSoft®, USA) package, with the level of significance set at *P* < 0.05.

## Results

The PBMCs of 115 CLL patients and 40 controls were tested for the presence of EBV-DNA with an aid of real-time PCR. A total of 62 (53.91%) CLL patients presented with a large number of EBV-DNA copies (more than 10 copies/*μ*L). The following three groups were identified on the basis of this criterion: (a) CLL patients whose PBMCs showed the presence of the EBV-DNA, i.e. the EBV(+) group, (b) CLL patients whose PBMCs lacked the EBV-DNA, i.e. the EBV(–) group and (c) healthy controls.

**Fig 1:**
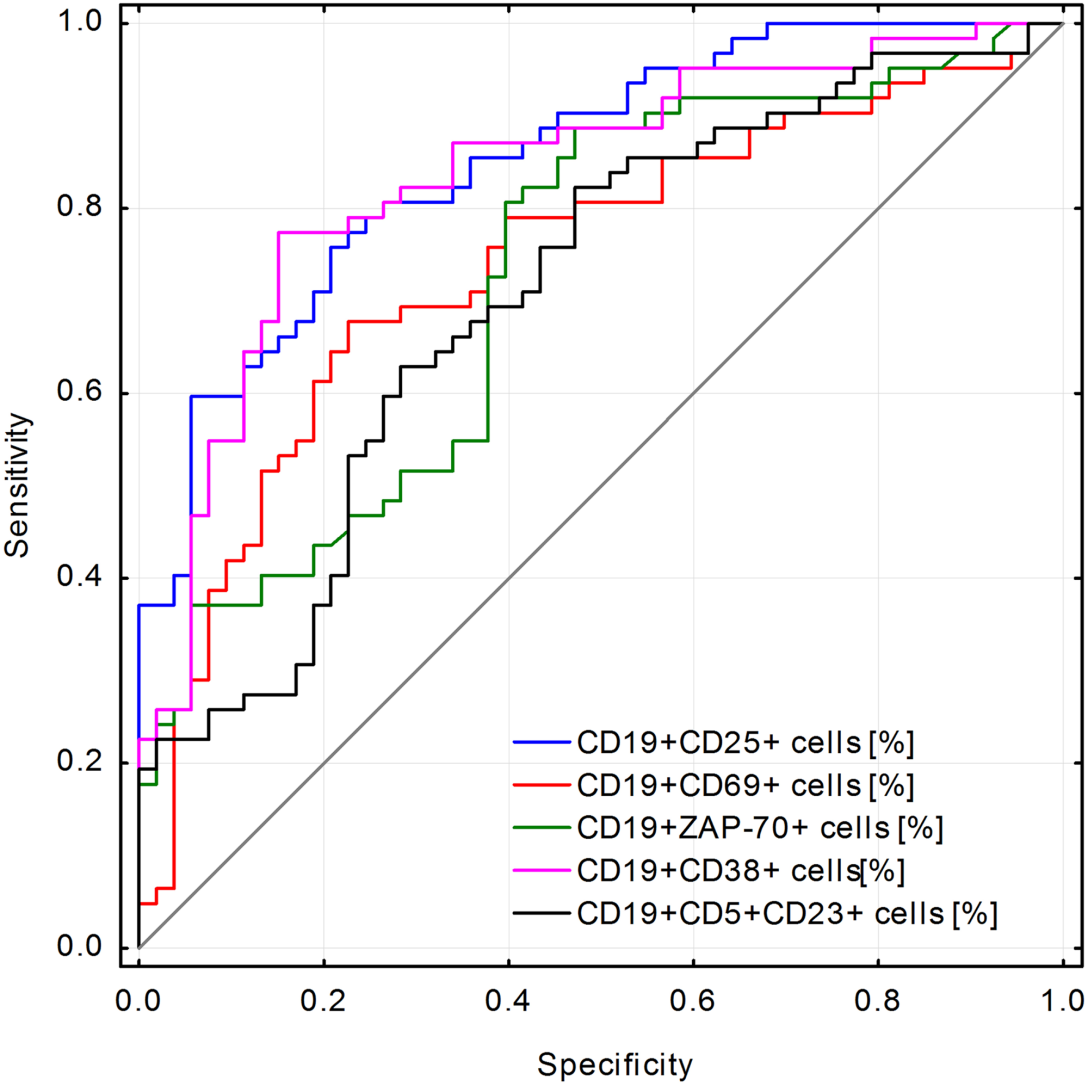
Comparison of the different immunological parameters found in peripheral blood for their ability to predict the presence of EBV. ROC curves were used to compare the sensitivity and specificity of immunological parameters (e.g. CD19+CD25+) in EBV(+) and EBV(–) CLL patients. The percentage of CD19+CD25+ cells in the peripheral blood was the most sensitive and specific parameter for determining a positive EBV reading (AUC = 0.854).

**Fig 2:**
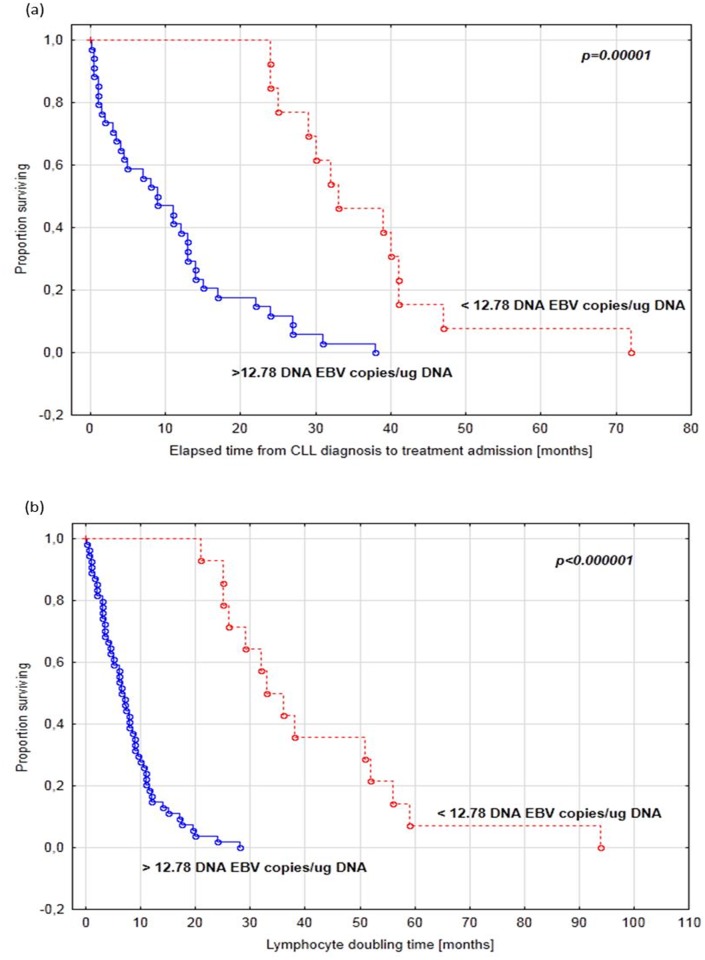
The time to first treatment and the probability of lymphocyte doubling-free survival depends on the EBV-DNA copy number in peripheral blood mononuclear cells (PBMCs) from chronic lymphocytic leukemia patients. (a) Kaplan-Meier curve illustrating the time to first treatment depending on the EBV-DNA copy number/μg DNA isolated from PBMCs; (b) Kaplan-Meier curve illustrating the probability of lymphocyte doubling-free survival depending on the EBV-DNA copy number/*μ*g DNA isolated from PBMCs.

**Fig 3:**
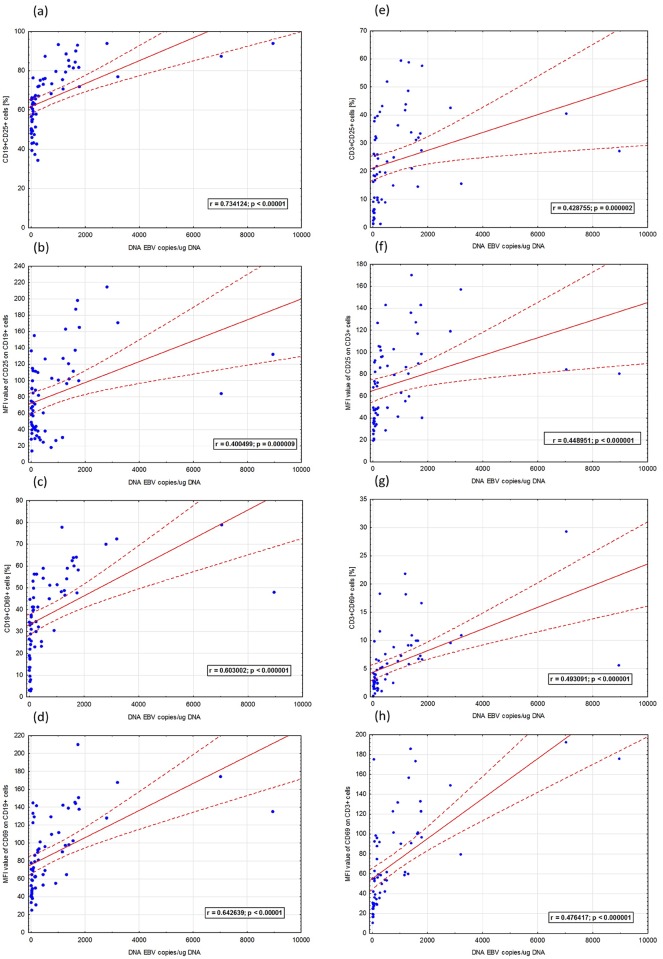
Statistically significant correlations between activated CD3+ T cells and CD19+ B cells in CLL patients and EBV-DNA viral load. (a) Positive correlation between the frequencies of CD19+CD25+ cells (%) and EBV-DNA copies/*μ*g DNA; (b) positive correlation between the mean fluorescent intensity (MFI) of CD25 on CD19+ cells (%) and EBV-DNA copies/*μ*g DNA; (c) positive correlation between the frequencies of CD19+CD69+ cells (%) and EBV-DNA copies/*μ*g DNA; (d) positive correlation between the MFI of CD69 on CD19+ cells (%) and EBV-DNA copies/*μ*g DNA; (f) positive correlation between the frequencies of CD3+CD25+ cells (%) and EBV-DNA copies/*μ*g DNA; (g) positive correlation between the MFI of CD25 on CD3+ cells (%) and EBV-DNA copies/*μ*g DNA; (h) positive correlation between the frequencies of CD3+CD69+ cells (%) and EBV-DNA copies/*μ*g DNA; (i) positive correlation between the MFI of CD69 on CD3+ cells (%) and EBV-DNA copies/*μ*g DNA.

In univariable analysis, among clinical and biochemical parameters of CLL patients both traditional and new prognostic factors associated with shorter time to first treatment turn out to be statistically significant (Table 1). We found that categorical variables such as the splenomegaly, trisomy 12, CD38, and positive anti-EBV EA IgA were associated with time to first treatment (Table 1). Among continuous variables lower hemoglobin, higher beta-2 microglobulin and LDH, higher CD19+CD25+ cells, CD19+CD69+ cells, CD3+CD69+ cells, CD19+CD5+CD38+ cells and CD19+CD5+CD23+ cells had significant association with time to treatment. Higher level of anti-EBV: EA IgG, EBNA IgG and VCA IgG were associated with shorter time to first treatment. Interestingly, EBV(+) patients had more than 7 times shorter time to first treatment comparing to EBV(-) patients. This phenomenon was confirmed during multivariable analysis. The multivariable Cox analysis identified the higher beta-2-microglobulin (HR = 1.43; p = 0.0033), enlarged spleen (HR = 4.70; p<0.0001), EBV presence (HR = 23.39; p<0.0001) and lower anti-EBV EBNA-1 IgM level (HR = 0.98; p = 0.0089) as independent predictors for shorter time to first treatment (Table 3a). Since the Cox proportional hazard regression model showed highly statistically significant association of EBV(+) with progression of illness, a ROC curve was drawn to test the variables predictive validity in EBV(+) CLL patients (Table 3b). As the area under the curve shows (Fig 1), CD19+CD25+ cells [%] in the peripheral blood parameter was the most sensitive and specific to determine EBV(+) (AUC = 0.854). Fig 2 presents a Kaplan-Meier curve illustrating the time to first treatment depending on the EBV-DNA copy number/*μ*g DNA isolated from PBMCs (Fig 2a) and a Kaplan-Meier curve illustrating the probability of lymphocyte doubling-free survival depending on the EBV-DNA copy number/*μ*g DNA isolated from PBMCs (Fig 2b). Table 4 presents the comparison between clinical and laboratory parameters in CLL EBV(+) patients, CLL EBV(–) patients and the study group. Table 5 presents an assessment of anti-EBV antibody concentrations in relation to the presence or absence of EBV-DNA copies in PBMCs of CLL patients and control group. Fig 3 presents statistically significant correlations between activated T CD3+ and B CD19+ cells in CLL patients.

**Table 3:** **(a).** Multivariable Cox Proportional Hazards Model for time to first treatment. **(b).** Receiver operating characteristic analysis to determine diagnostic accuracy in differentiation of patients with EBV(+) and EBV(-).

**(a)**				
**Parameter**	**Hazard Ratio**	**95% CI for hazard ratio**		**P value**
		**Lower**	**Upper**	
Beta-2-microglobulin [mg/dL]	1.43	1.12	1.83	0.0033
Anti-EBV EBNA-1 IgM [U/mL]	0.91	0.84	0.98	0.0089
EBV positive	23.29	7.7	70.39	<0.0001
Splenomegaly positive	4.70	2.20	10.01	<0.0001
**Parameter**	**Hazard Ratio**	**95% CI for hazard ratio**		**P value**
		**Lower**	**Upper**	
Beta-2-microglobulin [mg/dL]	1.45	1.15	1.82	0.0017
EBV positive	32.93	9.67	112.18	<0.0001
Splenomegaly positive	3.33	1.64	6.79	0.0009
Anti-EBV EA IgG positive	0.33	0.15	0.72	0.0055
**(b)**				
**Parameter**	**Prognostic value**	**Area under the ROC curve (AUC)**		**95%CI**
Beta-2-microglobulin (mg/dL)	2.63	0.742		0.65–0.83
Lactate dehydrogenase (U/L)	261	0.739		0.65–0.83
CD19+CD25+ cells [%] *	54.75	0.854		0.79–0.92
CD19+CD69+ cells [%] *	29.81	0.74		0.65–0.83
CD19+CD5+ZAP-70+ cells [%]*	6.31	0.726		0.63–0.82
CD19+CD5+CD38+ cells [%]*	10.74	0.838		0.76–0.91
CD19+CD5+CD23+ cells [%] *	75.59	0.707		0.61–0.80

* in the peripheral blood

**Table 4:** Comparison between clinical and laboratory parameters in CLL EBV(+) patients, CLL EBV(-) patients and the control group.

Parameter	Patients EBV(+)		Patients EBV(-)		Control group		Patients EBV(+) vs. EBV(-)		Patients EBV(+) vs. Control group		Patients EBV(-) vs. Control group	
	Mean ± SD	Median (range)	Mean ± SD	Median (range)	Mean ± SD	Median (range)	Z/t	*P* value	Z/t	*P* value	Z/t	*P* value
Age [years]	64.7±9.7	64.0 (39–89)	62.8±10.1	65.0 (38–79)	64.5±7.1	64.0 (53–79)	0.98	0.33	0.08	0.94	0.66	0.51
Elapsed time from CLL diagnosis to the treatment admission [months]	5.9±9.0	0.5 (0–38)	9.0±17.1	0 (0–72)	N/A	N/A	1.86	0.062	N/A	N/A	N/A	N/A
Observation period [months]	33.4±11.8	32.5 (10.5–72.0)	34.8±15.1	33.0 (14–80)	N/A	N/A	0.04	0.97	N/A	N/A	N/A	N/A
Lymphocytosis doubling time [months]	6.8±6.2	6.0 (0–28)	10.9±20.8	10 (0–94)	N/A	N/A	3.50	0.0005	N/A	N/A	N/A	N/A
Leukocytosis [x10^3 cells/μL]	37.4±21.3	29.1 (11.2–93.7)	36.8±25.0	30.5 (9.7–128.0)	7.0±1.4	7.0 (4.2–9.6)	0.64	0.52	6.69	<0.0001	6.55	<0.0001
Lymphocytosis [x10^3 cells/μL]	31.4±20.3	24.1 (8.9–87.5)	30.7±24.4	24.6 (6.2–124.0)	2.7±0.9	2.6 (1.4–5.2)	0.72	0.47	6.69	<0.0001	6.55	<0.0001
Hemoglobin [g/dL]	12.1±1.4	12.1 (9.0–15.7)	14.2±1.3	14.2 (9.9–16.8)	14.3±1.2	14.4 (12.5–16.9)	8.35	<0.0001	6.41	<0.0001	0.36	0.72
Platelets [x10^3 cells/μL]	155.8±54.6	146.0 (57.0–295.0)	196.9±55.2	194.0 (80.0–309.0)	279.0±57.0	281.5 (186.0–403.0)	4.0	0.0001	8.68	<0.0001	5.61	<0.0001
Beta-2-microglobulin [mg/L]	3.3±1.4	3.0 (1.3–8.1)	2.3±0.8	2.3 (1.1–5.9)	1.6±0.4	1.7 (1.1–2.3)	4.47	<0.0001	5.74	<0.0001	4.09	<0.0001
Lactate dehydrogenase [U/L]	354.3±143.0	323.5 (114.0–955.0)	250.8±94.5	229.0 (96.0–466.0)	157.0±28.0	155.5 (111.0–209.0)	4.41	<0.0001	6.33	<0.0001	4.33	<0.0001
CD19+CD25+ cells [%]*	66.4±15.6	66.4 (34.1–93.9)	41.5±17.2	44.1 (6.7–72.3)	2.8±1.5	2.8 (0.2–5.2)	6.52	<0.0001	6.69	<0.0001	6.55	<0.0001
MFI value of CD25 on CD19+ cells*	82.3±48.6	73.6 (13.9–213.9)	51.9±22.8	49.8 (12.8–105.9)	34.3±18.0	29.8 (6.4–70.1)	3.36	0.0008	4.42	<0.0001	2.99	0.003
CD19+CD69+ cells [%]*	38.4±19.3	39.6 (2.7–78.8)	22.5±16.1	20.9 (0.6–70.1)	1.0±0.8	0.8 (0.1–3.6)	4.45	<0.0001	6.66	<0.0001	6.38	<0.0001
MFI value of CD69 on CD19+ cells*	87.9±42.0	77.9 (24.6–209.9)	51.5±31.2	40.9 (21.6–177.9)	22.8±13.1	18.6 (7.3–59.9)	5.37	<0.0001	6.27	<0.0001	4.81	<0.0001
CD3+CD25+ cells [%]*	23.7±15.7	21.5 (1.2–59.4)	15.5±11.3	13.0 (0.6–45.1)	7.8±2.7	8.0 (1.1–11.5)	2.77	0.006	4.04	<0.0001	2.65	0.008
MFI value of CD25 on CD3+ cells *	71.4±37.2	68.5 (19.4–170.0)	50.6±32.6	41.2 (9.2–129.0)	41.6±17.1	41.5 (16.7–73.7)	3.12	0.002	3.15	0.002	0.44	0.66
CD3+CD69+ cells [%] *	5.9±5.6	4.1 (0.1–29.3)	3.2±2.8	2.4 (0.1–16.0)	1.7±0.8	1.8 (0.4–3.0)	3.20	0.001	4.29	<0.0001	1.94	0.053
MFI value of CD69 on CD3+ cells*	71.1±48.0	57.5 (10.6–192.2)	47.0±27.8	40.9 (10.2–120.9)	41.6±28.3	29.9 (10.4–113.9)	2.78	0.005	2.85	0.004	0.80	0.43
CD19+ZAP-70+ cells [%] *	20.9±13.5	18.1 (0.9–61.3)	10.9±9.5	5.9 (0.5–33.8)	N/A	N/A	4.17	<0.0001	N/A	N/A	N/A	N/A
CD19+CD38+ cells [%] *	35.7±26.4	33.8 (0.4–91.4)	8.0±14.9	1.6 (0.2–65.0)	N/A	N/A	6.23	<0.0001	N/A	N/A	N/A	N/A
CD19+CD5+CD23+ cells [%]*	82.9±10.6	86.5 (50.7–95.9)	73.8±13.0	74.8 (46.1–92.4)	N/A	N/A	3.82	0.0001	N/A	N/A	N/A	N/A
CD3+ cells [%]*	10.5±7.9	8.7 (1.2–33.4)	10.1±6.6	8.3 (1.0–28.6)	68.2±5.0	67.8 (61.5–78.6)	0.13	0.90	6.69	<0.0001	6.55	<0.0001
CD19+ cells [%]*	85.2±10.3	86.4 (51.5–97.8)	85.7±8.2	86.7 (67.0–98.3)	12.2±2.9	11.8 (7.8–16.6)	0.20	0.84	6.69	<0.0001	6.55	<0.0001
CD5+CD19+ cells [%]*	84.2±11.5	86.8 (54.9–99.6)	87.0±8.7	86.5 (70.0–99.7)	13.2±3.6	13.1 (5.7–19.6)	0.94	0.35	6.69	<0.0001	6.55	<0.0001
Serum IgA concentration [g/L]	1.7±1.2	1.4 (0.1–5.4)	1.2±0.7	1.1 (0.2–2.9)	2.4±0.8	2.6 (0.9–3.9)	2.05	0.041	2.96	0.003	4.82	<0.0001
Serum IgG concentration [g/L]	9.8±4.1	9.6 (3.5–19.4)	8.3±2.8	8.3 (3.2–14.7)	12.7±1.4	12.8 (10.1–15.5)	1.81	0.07	3.43	0.0006	5.31	<0.0001
Serum IgM concentration [g/L]	0.7±0.5	0.6 (0.04–3.1)	0.6±0.5	0.5 (0.05–2.2)	1.7±0.3	1.6 (1.2–2.2)	1.36	0.17	5.77	<0.0001	5.81	<0.0001

MFI, Mean Fluorescent Intensity;

N/A., not applicable;

* in the peripheral blood

**Table 5:** Serum anti-EBV antibody concentrations (i.e. IgG, IgM, IgA) in EBV(+) CLL patients, EBV(-) CLL patients, and the control group.

Antibody serum concentration [U/mL]	Patients EBV(+)		Patients EBV(-)		Control group		PatientsEBV(+) vs. EBV(-)		Patients EBV(+) vs. Control group		Patients EBV(-) vs. Control group	
	Mean ± SD	Median (range)	Mean ± SD	Median (range)	Mean ± SD	Median (range)	Z/t	*P* value	Z/t	*P* value	Z/t	*P* value
Anti-EBV EA IgA	51.5±113.5	6.7 (2.4–580.4)	5.9±5.2	4.8 (1.0–33.3)	3.7±1.7	3.7 (1.6–7.5)	2.91	0.004	3.83	0.0001	2.43	0.015
Anti-EBV EA IgG	87.1±105.6	38.1 (3.2–381.1)	16.0±20.3	8.2 (1.9–94.8)	8.5±4.5	8.2 (1.7–15.9)	5.60	<0.0001	4.78	<0.0001	0.60	0.55
Anti-EBV EA IgM	7.4±10.2	4.1 (1.4–62.7)	4.3±2.9	3.6 (0.8–14.2)	3.6±2.0	3.0 (1.0–7.3)	1.83	0.067	1.81	0.071	0.40	0.69
Anti-EBV EBNA-1 IgA	11.1±13.9	5.3 (1.2–78.5)	6.3±4.8	5.4 (1.5–30.4)	4.0±1.9	3.6 (1.2–7.0)	0.34	0.73	1.55	0.12	2.20	0.028
Anti-EBV EBNA-1 IgG	219.9±328.3	87.8 (22.5–1701.8)	60.7±32.5	55.7 (22.4–181.7)	63.0±26.5	55.8 (34.0–139.8)	3.18	0.001	1.65	0.10	0.62	0.53
Anti-EBV EBNA-1 IgM	7.6±5.0	6.0 (1.8–20.7)	4.9±2.3	4.8 (0.9–10.8)	4.9±1.7	4.7(2.2–7.8)	2.53	0.011	1.67	0.095	0.30	0.76
Anti-EBV VCA IgA	12.7±10.7	9.3 (1.9–50.9)	8.1±4.8	5.8 (2.0–19.8)	6.4±3.7	5.7 (1.4–13.7)	1.57	0.12	2.24	0.025	1.30	0.19
Anti-EBV VCA IgG	207.0±83.7	222.8 (30.3–352.8)	150.3±90.9	134.6 (23.8–358.1)	148.0±71.2	139.3 (33.4–274.9)	3.52	0.0004	2.97	0.003	0.15	0.88

## Discussion

A total of 20 CLL patients had more than 1000 EBV-DNA copies/*μ*g of PBMC DNA; the EBV-DNA copy number of the remaining patients ranged between 100 and 1000 (*N* = 22) or between 10 and 100 (*N* = 20). These results are similar to the data reported by Kimura et al. [12], according to whom the EBV-DNA copy number per *μ*g DNA extracted from PBMCs of patients with EBV-related lymphoproliferative disorders and infectious mononucleosis ranges between 10 and 10000. In contrast, the EBV-DNA copy number in most healthy controls and immunocompromised patients after organ transplantation did not exceed 10 copies/*μ*g DNA. Both Kimura et al. [12] and Stevens et al. [13] postulated that more than 100–1000 EBV-DNA copies/*μ*g DNA isolated from examined material are associated with clinical signs of EBV infection, manifesting as the chronic active EBV disease (CAEBV) or post-transplant lymphoproliferative disease. These findings and the results of our study point to a potential presence of a CLL subtype being associated with EBV infection. Moreover, an increase in the EBV-DNA copy number was documented in most of our patients during approximately 2-year follow-up.

We revealed the presence of EBV-DNA in PBMCs and isolated B lymphocytes in more than a half of our CLL patients. To the best of our knowledge, no previous studies distinguished between the CLL forms being associated with EBV infection or unrelated to this virus. The EBV-associated form of CLL seems to be characterized by more aggressive phenotype. We showed that the EBV-DNA copy number in PBMCs of patients with hepatomegaly or thrombocytopenia and individuals who required earlier implementation of treatment was significantly higher than that in the remaining individuals. A number of previous studies documented the role of EBV in induction of thrombocytopenia. While the presence of EBV in patients with infectious mononucleosis is usually associated with a slight decrease in platelet count, in the case of CAEBV, it can be associated with severe thrombocytopenia, anemia (usually of autoimmune origin), and splenomegaly (resulting from lymphocyte infiltration) or even liver failure [14, 15–16].

Moreover, we showed that the EBV-DNA copy number correlated significantly with serum concentrations of beta-2-microglobulin and LDH. As early as 1981, Ibsen et al. [17] revealed that the level of beta-2-microglobulin is at its highest during initial stages of infectious mononucleosis, and subsequently, within 3 weeks to 3 months after recovery, it normalizes to its baseline level. The fact that concentration of beta-2-microglobulin constitutes an established predictive factor in CLL patients may suggest that the elevated level of this protein is associated with EBV infection in at least some of the cases [18]. Furthermore, we revealed significant associations between other negative prognostic factors such as high cytoplasmic expression of ZAP-70 [19], surface expression of CD38 in leukemic cells [20], surface expression of CD23, CD25, and CD69 [21, 22], as well as unfavorable genetic mutations [23], and EBV-DNA copy number.

Tsimberidou et al. reported that 38% of CLL patients had evidence of EBV infection by in situ hybridization for EBV EBER1, a small noncoding RNA species [24]. Tarrand et al. [25] reported that LMP1 mRNA levels were higher in CLL patients than in healthy subjects (14% vs. 1% of healthy controls), suggesting that EBV late gene expression occurs at least in a subset of CLL cells. We demonstrate significant associations between viral load of EBV-DNA and various clinical and pathologic variables among CLL patients, including associations with progression and time to treatment. These findings are in line with conclusions made by Visco et al. [26] who postulated that EBV-DNA load at presentation is an independent predictor of overall survival in patients with CLL.

## Conclusions

In conclusion, more than a half of CLL patients presented with CLL EBV-DNA in their PBMCs, whereas no detectable amounts of genetic material for this pathogen were found in healthy controls. Greater EBV-DNA copy number was associated with shorter overall survival and time to progression in CLL patients. Positive correlation between EBV-DNA copy number and established unfavorable prognostic factors of CLL implies that increased EBV load in peripheral blood may predict poor clinical outcomes of CLL.
